# Human intestinal colonization by *Escherichia coli* ST4014 co-harboring *tet*(X4) and *bla*_NDM-1_ gene: a potential reservoir for antimicrobial resistance dissemination

**DOI:** 10.1128/spectrum.03336-25

**Published:** 2026-01-21

**Authors:** Huiqiong Jia, Shaocong Lu, Yuanyuan Jia, Yawen Yu, Yuye Wu, Danni Bao, Yingying Zhang, Jiehong Fang, Patrick Butaye, João Pedro Rueda Furlan, Mohamed Elhadidy, Dianelys Quiñones Pérez, Qing Yang, Zhi Ruan

**Affiliations:** 1 Department of Laboratory Medicine, The First Affiliated Hospital, Zhejiang University School of Medicine 71069, Hangzhou, China; 2 Zhejiang Key Laboratory of Clinical In Vitro Diagnostic Techniques, Hangzhou, China; 3 The First Clinical College, Chongqing Medical University, Chongqing, China; 4 Suzhou Medical College of Soochow University 74565, Suzhou, China; 5 Department of Clinical Laboratory, Sir Run Run Shaw Hospital, Zhejiang University School of Medicine 56660, Hangzhou, China; 6 Department of Clinical Laboratory, Sanmen People’s Hospital 647627 , Taizhou, Zhejiang, China; 7 Key Laboratory of Specialty Agri-products Quality and Hazard Controlling Technology of Zhejiang Province, College of Life Sciences, China Jiliang University 92270, Hangzhou, China; 8 Department of Infectious Diseases and Public Health, Jockey Club College of Veterinary, City University of Hong Kong 597149, Hong Kong, Hong Kong SAR, China; 9 Department of Pathobiology, Pharmacology and Zoological Medicine, Faculty of Veterinary Medicine, Ghent University 703523, Merelbeke, Belgium; 10 Department of Pharmaceutical Sciences, Health Sciences Center, Federal University of Paraíba 28097, João Pessoa, Paraíba, Brazil; 11 Center for Genomics, Helmy Institute for Medical Sciences, Zewail City of Science and Technology 359772, Giza, Egypt; 12 Biomedical Sciences Program, University of Science and Technology, Zewail City of Science and Technology 359772, Giza, Egypt; 13 Department of Bacteriology, Mycology and Immunology, Faculty of Veterinary Medicine, Mansoura University 158400, Mansoura, Egypt; 14 Healthcare-Associated Infections National Laboratory, Pedro Kourí Institute of Tropical Medicine 115350 , Havana, Cuba; 15 Key Laboratory of Precision Medicine in Diagnosis and Monitoring Research of Zhejiang Province, Hangzhou, China; 16 Zhejiang Provincial Engineering Research Center of Innovative Instruments for Precise Pathogen Detection, Hangzhou, China; Universidade de Sao Paulo, Sao Paulo, Brazil

**Keywords:** *Escherichia coli*, *tet*(X4), *bla*_NDM-1_, human gut colonization, antimicrobial resistance

## Abstract

This study aims to elucidate the genomic characteristics of three *Escherichia coli* strains isolated from stool specimens of healthy individuals co-carrying *tet*(X4) and *bla*_NDM-1_ gene, which confer resistance to tigecycline and carbapenems, respectively. Whole-genome sequencing (WGS) and bioinformatic analysis were conducted to identify the genomic characteristics. Fourteen *E. coli* strains belonging to the same sequence type (ST) 4014, comprising eleven strains retrieved from public databases and three strains from this study, were integrated into a phylogenetic analysis. Conjugation experiments were conducted to evaluate the transferability of the resistance plasmids. Three *E. coli* strains exhibited resistance to both tetracyclines and carbapenems, consistent with the presence of *tet*(X4) and *bla*_NDM-1_ genes in their genomes. All strains belonged to the rare ST 4014 and were identified in healthy individuals within a 1-week period. WGS revealed that tet(X4) and *bla*_NDM-1_ genes were each located on separate plasmids, both exhibiting 100% sequence identity to others previously reported in various bacterial species. Conjugation experiments confirmed the transferability of both *tet*(X4) and *bla*_NDM-1_-carrying plasmids. Phylogenetic analysis based on cgSNPs revealed limited genetic diversity among the three strains (2–6 SNPs), but substantial differences compared to 11 publicly available ST4014 strains (116–172 SNPs). This study reports *E. coli* ST4014 strains from healthy individuals harboring conjugative plasmids carrying *tet*(X4) and *bla*_NDM-1_ genes, conferring resistance to tigecycline and carbapenems, respectively. These findings highlight the silent spread of multidrug-resistant strains in community populations and emphasize the need for enhanced surveillance of antimicrobial resistance beyond clinical settings.

**IMPORTANCE:**

The emergence of *Escherichia coli* strains co-harboring *tet*(X4) and *bla*_NDM-1_ genes in healthy individuals represents a critical public health concern. These genes mediate resistance to tigecycline and carbapenems, two of the few remaining options for treating infections caused by multidrug-resistant gram-negative bacteria. The detection of clonally related ST4014 strains carrying conjugative plasmids encoding both resistance determinants highlights the potential for horizontal gene transfer and silent dissemination of dual-resistance plasmids in community settings. Such colonization among healthy individuals suggests that antimicrobial resistance may be spreading unnoticed beyond hospitals, driven by environmental or foodborne transmission routes. These findings emphasize the urgent need for integrated genomic surveillance and One Health-based interventions encompassing human, animal, and environmental reservoirs to prevent the expansion of high-risk resistance genes and safeguard the clinical efficacy of last-line antibiotics.

## OBSERVATION

Carbapenem-resistant *Escherichia coli* (CREC) mainly results from the production of carbapenem-hydrolyzing enzymes, especially New Delhi metallo-β-lactamase (NDM). Although CREC is primarily associated with clinical settings, strains from healthy individuals exhibit greater lineage diversity compared to those from clinical sources, suggesting a broader dissemination in non-clinical environments (1). The widespread occurrence of *bla*_NDM_ in CREC poses major challenges for antimicrobial therapy. Tetracycline derivatives, such as tigecycline, remain among the limited therapeutic options available for CREC infections (2). The discovery of mobile tigecycline resistance genes, such as *tet*(X) variants, in various species from clinical and environmental sources has weakened the effectiveness of this antimicrobial. In this study, we identified and characterized three *E. coli* strains isolated from stool specimens of healthy individuals that possess both *tet*(X4) and *bla*_NDM-1_ genes, demonstrating resistance to tigecycline and carbapenems.

In March 2021, strains SRY149, SRY157, and SRY206 were isolated from the stool samples of healthy individuals using Mueller-Hinton agar plates containing tigecycline (4 mg/L) at a tertiary hospital in Hangzhou, China. Strains were initially identified as *E. coli* using MALDI-TOF MS (Vitek MS system, bioMérieux, France) and then subjected to whole-genome sequencing. A detailed description of the materials and methods is provided in the supplemental material. *In silico* serotyping identified all strains as belonging to sequence type (ST) 4014 and serotype O88:H31. Strain SRY206 differed from SRY149 and SRY157 by six SNPs, whereas SRY149 and SRY157 differed by two SNPs from each other. Three strains were identified within 1 week, and the individuals carrying them exhibited a wide range of ages. No epidemiological link could be identified between the different carriers.

**Fig 1:**
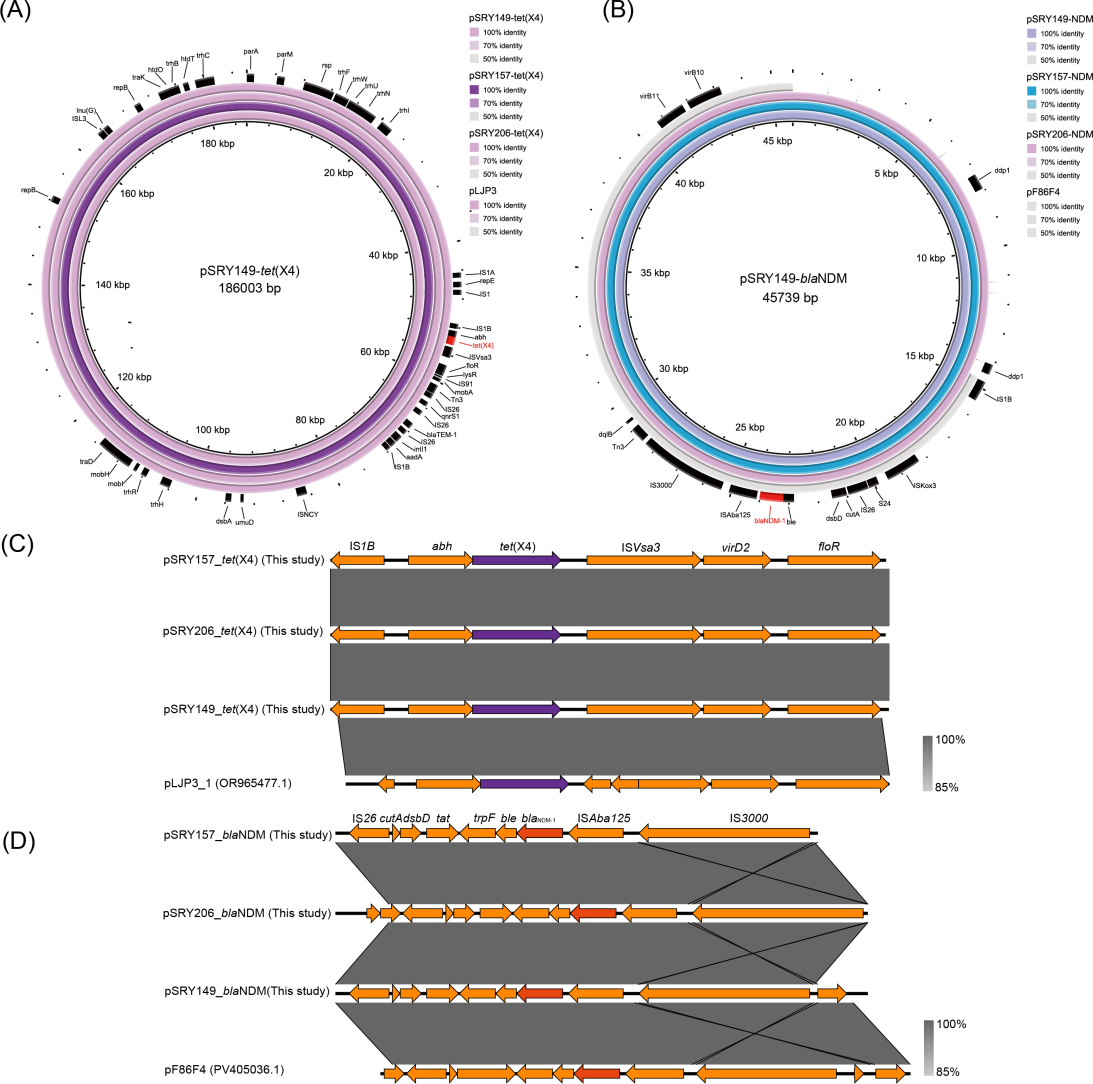
Comparative analysis of *tet*(X4) or *bla*_NDM-1_-carrying plasmids identified in this study. (**A**) Circular map comparing the *tet*(X4)-carrying plasmids identified in this study with related plasmids retrieved from public databases. (**B**) Comparative map of *bla*_NDM-1_-carrying plasmids, showing structural similarities and variations among the plasmids analyzed. (**C**) Genomic context of the *tet*(X4) gene identified in this study. The *tet*(X4) gene is highlighted in purple. (**D**) Genomic environment of the *bla*_NDM-1_ gene. The *bla*_NDM-1_ gene is shown in red.

Antimicrobial susceptibility testing results are summarized in Table S1. The strains possess a diverse array of antimicrobial resistance genes (ARGs). Strains SRY149 and SRY157 shared nearly identical ARGs [*tet*(X4), *tet*(A), *bla*_NDM-1_, *bla*_TEM-1B_, *bla*_CARB-2_, *floR*, *cmlA1*, *qnrS1*, *sul3*, and *lnu*(G)], while SRY206 harbored distinct ARGs. ARGs in SRY149, SRY157, and SRY206 strains were predominantly located on plasmids. The *tet*(X4) and *bla*_NDM-1_ genes identified in the three strains were located on two distinct plasmids. The *tet*(X4)-carrying plasmids, designated pSRY149-*tet*(X4) (186,003 bp), pSRY157-*tet*(X4) (186,831 bp), and pSRY206-*tet*(X4) (186,003 bp), were of comparable size and classified as IncHI1A/IncHI1B/IncFIA multi-replicon plasmids. The three *bla*_NDM-1_-harboring plasmids (pSRY149-*bla*_NDM_, pSRY157-*bla*_NDM_, and pSRY206-*bla*_NDM_) shared identical features, including a size of 45,739 bp and an IncX3 replicon type. The genetic environments of the *tet*(X4) gene in these plasmids were identical, consisting of structure IS*1B-abh-tet*(X4)-IS*Vsa3*(ΔIS*CR2*). Various genetic contexts of *tet*(X4) have been previously reported, with IS*CR2* or ΔIS*CR2* commonly associated with this gene (3). BLASTN analysis of the three *tet*(X4)-carrying plasmids against the NCBI RefSeq database revealed high sequence similarity with pLJP3_1 (OR965477.1), with 100% coverage and 100% identity observed for pSRY157-*tet*(X4) and pSRY206-*tet*(X4), and 100% coverage and 99.9% identity for pSRY149-*tet*(X4) (Fig. 1A and C). The three *bla*_NDM-1_-harboring plasmids (pSRY149-*bla*_NDM_, pSRY157-*bla*_NDM_, and pSRY206-*bla*_NDM_) were identical and carried the IS*3000*-IS*Aba125-bla*_NDM-1_-*ble-trpF* resistance module. High similarity was found with other *bla*_NDM-1_-containing plasmids from multiple bacterial species, including *E. coli*, *Klebsiella aerogenes*, *Klebsiella pneumoniae*, *Enterobacter hormaechei*, and *Citrobacter freundii*. Our *bla*_NDM-1_-harboring plasmids were identical to pF86F4 (PV405036.1) from *E. coli* and pKQ23-NDM1 (OQ230790.1) from *Klebsiella quasipneumoniae* (Fig. 1B and D). These findings suggest widespread dissemination of this *bla*_NDM-1_-carrying plasmid type among diverse bacterial taxa.

The *tet*(X4)-carrying *E. coli* strains have been frequently identified in livestock (particularly pigs and poultry), meat products, and related environments across multiple regions in China (4). Three *E. coli* strains collected from pigeons in China were found to co-harbor *tet*(X4), *mcr-1*, and *bla*_NDM-5_ genes. The genes *tet*(X4) and *bla*_NDM-5_ were located on transferable plasmids**,** whereas *mcr-1* was encoded chromosomally (5). In this study, the conjugation frequencies of the *tet*(X4)-carrying plasmids ranged from 1.09 × 10^−6^ to 1.57 × 10^−4^, while those of the *bla*_NDM-1_-harboring plasmids ranged from 3.43 × 10^−7^ to 2.29 × 10^−5^. Recent research has described the first evidence of a *tet*(X3)-harboring plasmid capable of horizontal gene transfer across bacterial orders, specifically from *Pseudomonadales* (e.g., *Acinetobacter* spp.) to *Enterobacterales* (e.g., *Enterobacteriaceae*) (6). The emergence of multidrug-resistant *E. coli* strains carrying transferable plasmids with various ARGs in healthy individuals is concerning, as these strains are frequently not covered by standard surveillance systems. Particularly worrying is the occurrence of strains that carry ARGs but do not exhibit a resistant phenotype (7, 8). Such strains may contribute to the silent dissemination of ARGs and hinder antimicrobial resistance surveillance. As such, they constitute a major public health threat by facilitating the unnoticed spread of resistance within the community and hospital environments.

In this study, we report on *E. coli* strains co-harboring *tet*(X4) and *bla*_NDM-1_ genes in healthy individuals, indicating unnoticed colonization by bacteria resistant to tigecycline and carbapenems. Given that these antimicrobial agents represent last-line options for treating severe infections caused by multidrug-resistant gram-negative pathogens, the emergence and potential spread of such dual-resistant strains pose a major threat to effective antimicrobial therapy. These findings highlight the urgent need for expanded surveillance beyond healthcare settings to detect and control the dissemination of high-risk ARGs with pandemic potential.

## Data Availability

The genome sequences of *Escherichia coli* SRY149, SRY157, and SRY206 have been deposited in the National Center for Biotechnology Information under GenBank accession numbers CP173239–CP173253.

## SUPPLEMENTAL MATERIAL

Supplemental material spectrum.03336-25-s0001.docx Supplemental methods and Table S1.

ASM does not own the copyrights to Supplemental Material that may be linked to, or accessed through, an article. The authors have granted ASM a non-exclusive, world-wide license to publish the Supplemental Material files. Please contact the corresponding author directly for reuse.
