# Supplementary material for: Human intestinal colonization by Escherichia coli ST4014 co-harboring tet(X4) and blaNDM-1 gene: a potential reservoir for antimicrobial resistance dissemination
Source: Microbiol Spectr. 2026 Jan 21;14(3):e03336-25. doi: 10.1128/spectrum.03336-25 (PMC12955429; doi:10.1128/spectrum.03336-25)
Supplement: Supplemental material — Supplemental methods and Table S1. [file spectrum.03336-25-s0001.docx]

Supplementary Materials

Human intestinal colonization by *Escherichia coli* ST4014 co-harboring *tet*(X4) and *bla*_NDM-1_ gene: a potential reservoir for antimicrobial resistance dissemination

Huiqiong Jia^1,2,#^, Shaocong Lu^3, #^, Yuanyuan Jia^4, #^, Yawen Yu^5^, Yuye Wu^5^, Danni Bao^6^, Yingying Zhang^5^, Jiehong Fang^7^, Patrick Butaye^8,9^, João Pedro Rueda Furlan^10^, Mohamed Elhadidy^11,12,13,*^, Dianelys Quiñones Pérez^14,*^, Qing Yang^1,2,*^, Zhi Ruan^5,15,16,*^

*^1^Department of Laboratory Medicine, The First Affiliated Hospital, Zhejiang University School of Medicine, Hangzhou, China.*

*^2^Zhejiang Key Laboratory of Clinical In Vitro Diagnostic Techniques, Hangzhou, China;*

*^3^The First Clinical College, Chongqing Medical University, Chongqing, China.*

*^4^Suzhou Medical College of Soochow University, Suzhou, China.*

*^5^Department of Clinical Laboratory, Sir Run Run Shaw Hospital, Zhejiang University School of Medicine, Hangzhou, China*

*^6^Department of Clinical Laboratory, Sanmen People's Hospital, Taizhou, Zhejiang, China.*

*^7^Key Laboratory of Specialty Agri-products Quality and Hazard Controlling Technology of Zhejiang Province, College of Life Sciences, China Jiliang University, Hangzhou, China.*

*^8^Department of Infectious Diseases and Public Health, Jockey Club College of Veterinary, City University of Hong Kong, Hong Kong, Hong Kong SAR, China.*

*^9^Department of Pathobiology, Pharmacology and Zoological Medicine, Faculty of Veterinary Medicine, Ghent University, Merelbeke, Belgium.*

*^10^Department of Pharmaceutical Sciences, Health Sciences Center, Federal University of Paraíba, João Pessoa, Paraíba, Brazil.*

*^11^Center for Genomics, Helmy Institute for Medical Sciences, Zewail City of Science and Technology, Giza, Egypt.*

*^12^Biomedical Sciences Program, University of Science and Technology, Zewail City of Science and Technology, Giza, Egypt.*

*^13^Department of Bacteriology, Mycology and Immunology, Faculty of Veterinary Medicine, Mansoura University, Mansoura, Egypt.*

*^14^Healthcare-Associated Infections National Laboratory, Pedro Kourí Institute of Tropical Medicine, Havana, Cuba.*

*^15^Key Laboratory of Precision Medicine in Diagnosis and Monitoring Research of Zhejiang Province, Hangzhou, China.*

*^16^Zhejiang Provincial Engineering Research Center of Innovative Instruments for Precise Pathogen Detection, Hangzhou, China.*

^*^To whom correspondence should be addressed:

Zhi Ruan, r_z@zju.edu.cn;

Qing Yang, yq721227@zju.edu.cn;

Dianelys Quiñones Pérez, dianymariam@gmail.com;

Mohamed Elhadidy, melhadidy@zewailcity.edu.eg.

^#^These authors contributed equally to this work.

Key words: *Escherichia coli*, *tet*(X4), *bla*_NDM-1_, human gut colonization, antimicrobial resistance.

**MATERIALS AND METHODS**

**Clinical strains**

Strains SRY149, SRY157, and SRY206 were obtained from stool specimens of male patients at the same health center as follows: strain SRY149 was isolated from a 29-year-old male on March 22, 2021; strain SRY157 was recovered from a 39-year-old male on March 23, 2021; and strain SRY206 was obtained from a 35-year-old male on March 26, 2021.

Antimicrobial susceptibility testing (AST)

The antimicrobial susceptibility was evaluated to 27 antimicrobials (Table S1) using the Thermo Fisher Scientific Gram-negative minimum inhibitory concentration (MIC) plate GN4F. For antimicrobials not included in commercial susceptibility testing kits, including omadacycline, eravacycline, and colistin, the broth microdilution method was employed to determine the MICs. Clinical breakpoints for tigecycline and omadacycline were interpreted according to criteria established by the U.S. Food and Drug Administration (FDA), while those for eravacycline were determined according to EUCAST version 12.0. Interpretation of susceptibility for all other antimicrobial agents was performed in compliance with CLSI M100 (34th edition).

Whole-genome sequencing and bioinformatic analysis

Genome sequencing was performed using the Illumina HiSeq X10 (Illumina, San Diego, CA, USA) with the 150 bp paired-end protocol and long-read Oxford Nanopore sequencer (Nanopore, Oxford, UK). The sequences were hybrid assembled with Unicycler (v.0.4.8) (1). The BacWGSTdb 2.0 (2) server was used to conduct bioinformatics analysis of the sequences, including the identification of ARGs, plasmid replicons, and *in silico* multilocus sequence typing (MLST). The phylogenetic analysis was performed using Gubbins (v.3.3.4) (3) and Snippy (v.4.6.0). The visualization was carried out using iTOL (v.5) (4). Easyfig (v2.2.5) was used for aligning linear plasmid sequences. *In silico* serotype prediction of the three *E. coli* strains was carried out using the SerotypeFinder 2.0 web server (5).

Conjugation experiments

The transferability of plasmids containing *tet(*X4) and *bla*_NDM-1_ was evaluated using conjugation experiments. The *tet(*X4) and *bla*_NDM-1_-positive *E. coli* served as the donor, while *E. coli* EC600 functioned as the recipient. Transconjugants were selected on MHA plates supplemented with tigecycline (4 mg/L), meropenem (4 mg/L), and rifampicin (200 mg/L). Conventional polymerase chain reactions confirmed the presence of *tet(*X4) and *bla*_NDM-1_ genes in transconjugants.

Table S1. Antimicrobial susceptibility of *E. coli* ST4014 strains used in this study

| Strains | MIC (μg/mL) | | | | | | | | | | | | | | | | | | | | | | | | | | |
| --- | --- | --- | --- | --- | --- | --- | --- | --- | --- | --- | --- | --- | --- | --- | --- | --- | --- | --- | --- | --- | --- | --- | --- | --- | --- | --- | --- |
|  | TET | MIN | TGC | ERC | OMG | ETP | DOR | IPM | MEM | PIP | AMP | FAZ | CAZ | CRO | FEP | LEV | CIP | GEN | AMI | AZT | TOB | CST | P/T | T/C | SXT | A/S | NIT |
| SRY149 | >8 | >8 | >8 | 8 | 16 | >8 | >4 | 8 | 8 | >64 | >16 | >16 | >16 | >32 | 32 | <1 | <0.5 | <2 | <8 | <1 | <2 | <0.06 | >128/4 | >64/2 | >4/76 | >16/8 | <32 |
| SRY157 | >8 | >8 | >8 | 8 | 16 | >8 | >4 | >8 | >8 | >64 | >16 | >16 | >16 | >32 | 16 | <1 | <0.5 | <2 | <8 | <1 | <2 | <0.06 | >128/4 | >64/2 | >4/76 | >16/8 | <32 |
| SRY206 | >8 | >8 | >8 | 8 | 32 | >8 | >4 | 8 | >8 | >64 | >16 | >16 | >16 | >32 | 32 | <1 | <0.5 | <2 | <8 | <1 | <2 | <0.06 | >128/4 | >64/2 | >4/76 | >16/8 | <32 |

TET: Tetracycline, MIN: Minocycline, TGC: Tigecycline, ERC: Eravacycline, OMG: Omadacycline, ETP: Ertapenem, DOR: Doripenem, IPM: Imipenem, MEM: Meropenem, PIP: Piperacillin, AMP: Ampicillin, FAZ: Cefazolin, CAZ: Ceftazidime, CRO: Ceftriaxone, FEP: Cefepime, LEV: Levofloxacin, CIP: Ciprofloxacin, GEN: Gentamicin, AMI: Amikacin, AZT: Aztreonam, TOB: Tobramycin, CST: Colistin, P/T: Piperacillin/Tazobactam, T/C: Ticarcillin/Clavulanic Acid, SXT: Trimethoprim/Sulfamethoxazole, A/S: Ampicillin/Sulbactam, NIT: Nitrofurantoin.

REFERENCES

1. Wick RR, Judd LM, Gorrie CL, Holt KE. 2017. Unicycler: resolving bacterial genome assemblies from short and long sequencing reads. PLoS computational biology 13:e1005595.

2. Feng Y, Zou S, Chen H, Yu Y, Ruan Z. 2021. BacWGSTdb 2.0: a one-stop repository for bacterial whole-genome sequence typing and source tracking. Nucleic Acids Research 49:D644-D650.

3. Croucher NJ, Page AJ, Connor TR, Delaney AJ, Keane JA, Bentley SD, Parkhill J, Harris SR. 2015. Rapid phylogenetic analysis of large samples of recombinant bacterial whole genome sequences using Gubbins. Nucleic acids research 43:e15-e15.

4. Letunic I, Bork P. 2024. Interactive Tree of Life (iTOL) v6: recent updates to the phylogenetic tree display and annotation tool. Nucleic acids research 52:W78-W82.

5. Joensen KG, Tetzschner AM, Iguchi A, Aarestrup FM, Scheutz F. 2015. Rapid and easy in silico serotyping of Escherichia coli isolates by use of whole-genome sequencing data. Journal of clinical microbiology 53:2410-2426.
